# Russian Biodiversity Collections: A Professional Opinion Survey

**DOI:** 10.3390/ani13243777

**Published:** 2023-12-07

**Authors:** Elena V. Alpeeva, Natalia P. Sharova, Konstantin S. Sharov, Ekaterina A. Vorotelyak

**Affiliations:** Koltzov Institute of Developmental Biology of Russian Academy of Sciences, 26 Vavilov Street, 119334 Moscow, Russia; alpeeva_l@mail.ru (E.V.A.); npsharova@bk.ru (N.P.S.);

**Keywords:** biodiversity collection, biodiversity centre, biobank, biological conservation, extinction, wildlife restoration, endangered species

## Abstract

**Simple Summary:**

The rate of human population growth continues to increase, with a growing adverse anthropogenic influence on the biosphere. This creates a completely new evolutionary challenge to animal species and new research tasks for bioconservation science. Biocollections are very helpful in bioconservation in this situation. We studied the professional opinions of bioconservation specialists on the future of creating/maintaining biocollections and biocollection networks in Russia. There is a significant degree of concordance among them about the necessity to unite biocollections in networks. This may provide several important opportunities: the ability to cross-check in research, the simplicity of access, redundancy in storing specimens, and effective data curation. We show the success and deliberate on the future potential of our scientific institution in developing and sustaining four large biocollections. These biocollections may become a basis for a national biodiversity centre.

**Abstract:**

Biodiversity collections are important vehicles for protecting endangered wildlife in situations of adverse anthropogenic influence. In Russia, there are currently a number of institution- and museum-based biological collections, but there are no nation-wide centres of biodiversity collections. In this paper, we report on the results of our survey of 324 bioconservation, big-data, and ecology specialists from different regions of Russia in regard to the necessity to create several large national biodiversity centres of wildlife protection. The survey revealed specific goals that have to be fulfilled during the development of these centres for the protection and restoration of endangered wildlife species. The top three problems/tasks (topics) are the following: (1) the necessity to create large national centres for different types of specimens; (2) the full sequencing and creation of different “omic” (genomic, proteomic, transcriptomic, etc.) databases; (3) full digitisation of a biodiversity collection/centre. These goals may constitute a guideline for the future of biodiversity collections in Russia that would be targeted at protecting and restoring endangered species. With the due network service level, the translation of the website into English, and permission from the regulator (Ministry of Science and Higher Education of Russian Federation), it can also become an international project.

## 1. Introduction

### 1.1. Problem Overview

Biodiversity collections are significant instruments of biological conservation, wildlife restoration, and research in situations of climatic change, deforestation, carbonisation, waste accumulation, including the plastification of water reservoirs, and other anthropogenic activities [1,2,3]. By the second decade of the twenty-first century, only small amounts of truly “wild nature” remained on the planet. Humans transform nature to a degree when artificial infrastructure is embedded in natural biotopes and biocoenoses and sometimes “melted” with them.

The adverse anthropogenic influence causes the shrinking of animal habitats, species extinction, and biodiversity loss [4,5]. Humans have been responsible for the extinction of around 900 vertebrate species since the sixteenth century (of them, more than half were eradicated in the twentieth to twenty-first centuries) [6,7,8,9,10].

Not only do *homo sapiens* influence ecosystems by themselves and cause species to become extinct, they also cause a variety of species to interfere with others. To date, humans have introduced around 6800 invasive animal species to different parts of the world: i.e., they have relocated them from one part of the planet to another, sometimes unintentionally and sometimes on purpose [9]. In many cases, invasive animal species drive native species out of a given area by supplanting them or feeding on them and, sometimes, even changing biocoenoses beyond recognition [7]. Invasive species may proliferate excessively in the absence of their natural enemies in new living territories. This may lead to a direct substantial hazard to biodiversity, which often causes panzoonotics (animal analogues of pandemics) and may dramatically affect wild species [11,12,13,14]. Additionally, panzoonotics may easily give rise to outbreaks of novel human emerging infectious diseases (EIDs). There are two well-known examples demonstrating this [15,16].

First, the growth of the human population has caused the even faster growth of rat and mouse populations, which now number approximately 8 billion each, just like *homo sapiens*, as well as the street pigeon (feral pigeon) population, which consists of up to 0.5 billion specimens [16]. Due to their proximity to humans, these rodents and birds have become frequent causes of outbreaks as intermediary hosts for EID agents.

Second, over the last three decades, *homo sapiens* have bred enormous populations of the domesticated chicken, *Gallus gallus domesticus*. Now, chickens number around 24 billion specimens on the planet, as *homo sapiens* use this species as one of their main foods. During the H5N1 avian influenza epidemics in China and Southeast Asia in 1997–2005, chickens were the main intermediary hosts for the virus, which switched to them from several wild bird species [17]. Chickens were not bred in incubators in this region. They lived freely in paled yards where wild geese, jays, common mynas, and crows had unrestricted access as they fed on the chickens’ food. In turn, infected chickens began to infect wild birds. In some regions, huge colonies were wiped out. As a result, humans had to eradicate the largest part of the chicken population in Asia to stop the great bird panzootic.

**Figure 1:**
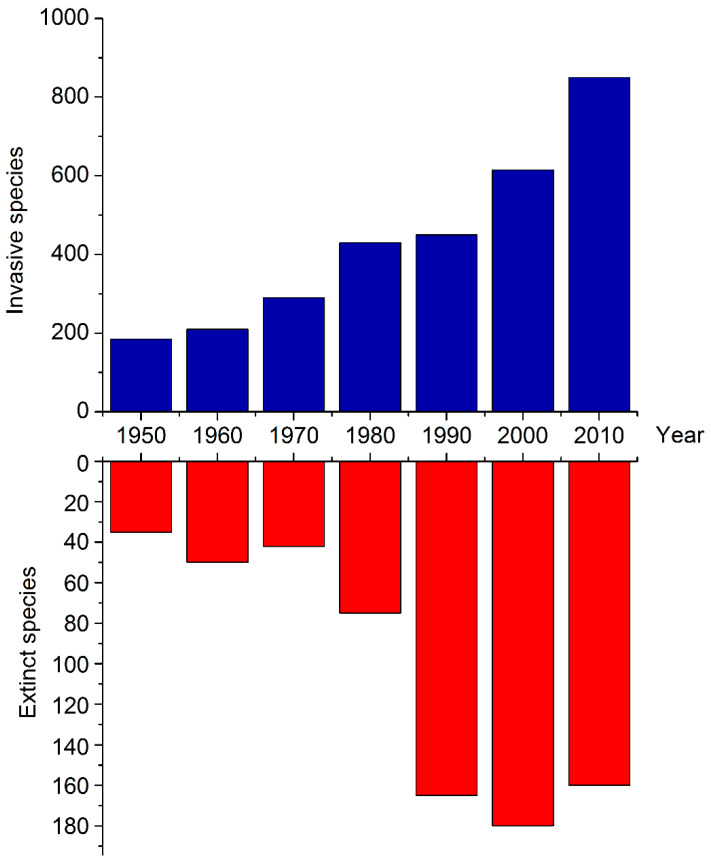
Vertebrate species’ extinction and invasion caused by humans during the last seventy years. Based on the data provided in [18,19].

Figure 1 demonstrates the dynamics of species extinction and invasion caused by anthropogenic influence.

The main ways in which humans influence the population sizes of animal species are as follows:

(1) The destruction of ecosystems and biogeocoenoses due to the founding of settlements, such as mega-cities in the extreme case, and their infrastructure (national electrical networks, roads, pipelines, etc.);
(2) Terraforming: cutting down forests, changing river beds, making water reservoirs, building artificial islands, etc., to generate spacious agricultural grounds or accommodate more people, including underwater lands and coastlines;
(3) The introduction of domesticated animals and plants (invasive species) into ecosystems and biogeocoenoses that cannot integrate them and, thus, are destroyed;
(4) The use of genetically modified husbandry species for food or agricultural forage;
(5) The induction of climate change due to the combustion of fossil fuels with the consequent greenhouse effect;
(6) The poorly deliberated use of alternative sources of energy;
(7) The wide use of wild food because of the augmented demand for traditional kitchens in the Far East, Southeast Asia, Latin America, and Africa;
(8) Chemical and biological pollution, including the carbonisation of the planet;
(9) The generation of huge masses of unprocessed waste; e.g., the plastification of the world’s oceans creates a perfect basis for the growth of bacterial colonies and damages thermohaline circulation, which begets a malfunction in sustaining the climate of aquatic forms of life.

About 68 percent of the vertebrate animal biomass has disappeared since 1970 [11]. There are estimations that by 2100, *homo sapiens* may place 4 to 5 million species of the 8–9 million species remaining on the Earth now on the edge of extinction. This is sometimes called the “sixth great extinction of species” [10,12,13,14]. The line between “nature” and “culture” has never been as blurred as it is now [15,16,17].

The anthropogenic influence causes such a high level of species extinction mainly due to the dramatic influence on planetary processes and biotopes.

Biodiversity collections cannot diminish the rates of animal species extinction caused by anthropogenic factors, but they can provide ways to restore the populations of some species through the following mechanisms:

(1) The direct conservation of genetic material;
(2) Extensive scientific research that may lead to finding new means to restore species populations [20].

### 1.2. International Experience in Creating Biocollections

Biodiversity collections may be subdivided into the following collection types:

(1) Collections of lifeless forms suitable only for research, e.g., fossils, herbaria, entomology, ichthyology, herpetology, ornithology, and mammal collections;
(2) Collections of lifeless forms suitable for both research and biota restoration, e.g., seed collections;
(3) Collections of live forms, e.g., cell collections, microbial collections, cryogenic banks of biological material (biobanks);
(4) Information collections that do not contain biological samples but only information about them [20].

The collections of the first three types can (in real life—must) be supplemented with electronic databases for their accessibility to the scientific community, business entities, and other concerned parties [21]. A typical modern biocollection often possesses the instruments for “omic” investigations (genomics, proteomics, transcriptomics, etc.) and radioisotope, X-ray phase, X-ray structure, chromatography–mass spectrometry, and other analyses [22]. Photography, video recordings (e.g., recording of cell processes or recording of the growth of a specimen), and tomography are widely used in biocollections to image specimens [20]. Different types of information about a specimen may be sorted, analysed, rendered to a human-friendly view, and stored in digital form using network protocols [23].

There is a discrepancy in distinguishing between biocollections and biobanks. Some researchers suggest neglecting any linguistic or semantic differences between these two terms [24]. Others conceive of a biobank as a biocollection at the national scale [25]. The third group proposes considering human biological materials to belong to a biobank, whereas non-human species belong to a collection [26]. The fourth group supposes that a biobank can provide or lease biological material just like a bank lends money, while a biocollection only stores and/or processes samples and the corresponding data [27]. In our work, we follow the fourth approach. We define a biobank as a collection of animal or human biological materials that are stored, analysed, and researched and can be provided for various purposes on a free or commercial basis any time the material is needed. Therefore, in this understanding, the notion of a biobank is a part of the notion of a collection.

An important advantage of a biocollection is its accessibility to external researchers via a file-transfer protocol (FTP) route, the Internet, or private networks [28]. For instance, genetic biobanks can be integrated into international databases such as GenBank [29]. Big-data computing, artificial intelligence, and machine-learning algorithms play important roles in genotype studies and phenotype characterisation [30,31]. Several state-of-the-art collections use AI (artificial intelligence) technologies allowing the detection of correlations between morphology and phylogeny or between morphology and ecologic perturbations that have influenced biota in a given geographical zone [32].

To sum up, biocollections are very important today. With their use, one can meet the challenges of different scientific and other fields, including performing taxonomic analysis to model biological processes and the behavioural patterns of animals.

**Figure 2:**
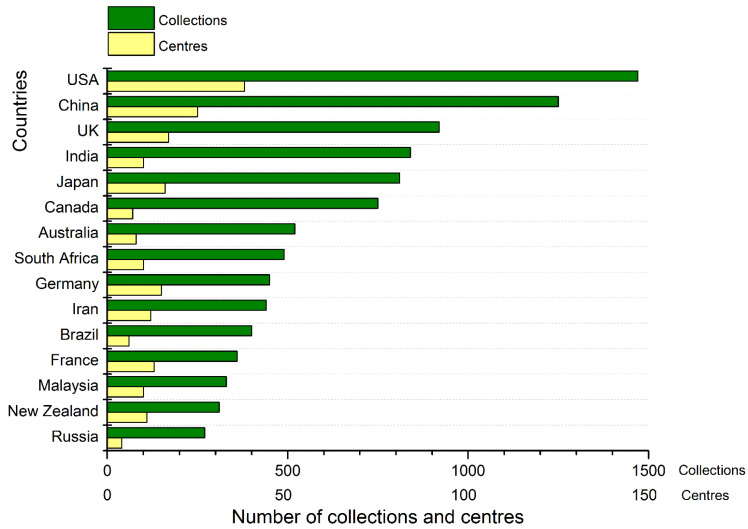
Number of documented biodiversity collections and centres important for bioconservation, agriculture, medicine, veterinary, and scientific research in fifteen chosen countries (as of 2021). Based on data provided in the studies [34,35,36].

In countries where biocollections have been developed for a considerably long time (thirty–fifty years), large nation-wide biodiversity centres have appeared [33]. Figure 2 shows the number of documented biocollections and large biodiversity centres in several countries, which are significant for bioconservation, agriculture, medicine, veterinary, and scientific research [34,35,36]. Large centres are of national and international importance. They can contain millions of specimens [35]. Important international collections and centres contain information in digital form; many of them also store metadata [36]. Around one-third of North American collections provide external online access to them [36].

A crucial step is the uniting of biodiversity collections and centres in networks. This may provide several important opportunities:

(1) The ability to cross-check in research;
(2) The simplicity of access;
(3) Redundancy in storing specimens;
(4) Effective data curation [37].

Data curation for many specimens is necessary even in the best biocollections, as, usually, information about a specimen may not be abundant [38]. In addition, progress in the development of technology, methods, and tools may eventually provide additional possibilities for analysis and effective data curation using appropriate algorithms [39].

The Pan American network (Extended Specimen Network, ESN) can serve as an example of a national network of biocollections. Its development commenced in 2011, and it includes the records of some 65 million specimens as of now [40]. By 2030, it is predicted to include the records of 1.2 billion specimens [40]. 

In the most advanced modern biocollections, one can trace a paradigmatic shift from the depositing and analysis of separate specimens to the storage of information about biotic associations [36]. Thus, the “omic” data about a species will be supplemented with meta-information about the following:

(a) Its habitat(s) (including geographic information system data);
(b) A typical ecological environment;
(c) Biogeocoenosis in which specimens of this species usually live;
(d) The behavioural patterns of animals;
(e) Organic and inorganic matter and forces (including climate and weather patterns in the habitat);
(f) Common food chains;
(g) Different organisms that form biotic associations with the species analysed (e.g., parasites, symbiotes, endophytes, epiphytes) [36].

Such an approach may open new possibilities in research. Studying population markers and characteristics can reshape our views on some evolutionary events, e.g., population bottlenecks, the contraction and expansion of habitats, transfer to another type of food, etc. [34]. More specifically, the genetic approach to studying speciation has developed largely due to success in creating a database of standardised genetic markers of different forms of life, such as Boldsystems [41]. Some local collections also provide sample information, e.g., the collection of PhMr. Tibor Weisz in Sarisske Museum Bardejov, Slovakia [42].

Table 1 compares three well-known international biodiversity centres. The comparison shows that biodiversity centres may have completely different tasks and technical specifications, but all of them have similar digitisation algorithms and enable easy online access to their databases for external research teams.

Biobanks, as a part of biocollections, are acquiring increasing importance for medical and veterinary purposes [47]. Their main medical and veterinary applications are transplantation, blood transfusion, and in vitro fertilisation procedures. In addition, they give an opportunity to perform genome-wide association studies (GWAS) [48]. An example of GWAS application is tracing the migration routes and interspecies genetic relations, i.e., studying the predisposition of a species to a given set of diseases [48].

We also witness a tendency to unite biobanks in networks. A prominent recent example is the Global Biobank Meta-Analysis Initiative, which combined digital data from twenty-four national cryobiobanks representing fifteen countries [49]. In this initiative, around 2.2 million genotype samples with approximately 70 million genetic variants have already been studied [50]. Thirteen diseases are currently under a comprehensive investigation in this network [37].

Important experience may be gained from the international biocollection networks that have been managed by US universities, scientific institutions, and societies for a long time, e.g., Network Integrated Biocollections Alliance (NIBA) [51] and Biodiversity Collections Network or Extended Specimen Network (BCN or ESN) [52]. They encompass more than one billion specimens. Both projects are rapidly developing. They offer a variety of research opportunities:

(1) Scientific discovery;
(2) Seamless data integration and attribution among different biocollections;
(3) The completion and improvement of digitised data;
(4) The ability to fill gaps in biodiversity data;
(5) The building and strengthening of international collaboration in bioconservation;
(6) The creation of an advanced specimen identification system;
(7) The development of new protocols for collecting underrepresented taxa;
(8) The provision of equal opportunities for small collections, either regional or personal;
(9) The provision of educational venues and capabilities;
(10) The strengthening of multidisciplinary work in bioconservation, biocollections, and big data.

The NIBA and ESN have caused a shift in our understanding of biocollections from separate specimens towards dynamic repositories of interconnected resources enriched by the study over time. They will help us to understand the organisms’ growth, diversification, and their interaction with one another, as well as how climate change and the anthropogenic influence may affect biotic associations.

### 1.3. Biocollections in Russia

Russia can utilise the rich international experience in creating and maintaining biocollections obtained during the last thirty–forty years. Its variety of climatic zones, big area, and many endemic taxa make Russia important in the context of biocollections. Likewise, it is important to utilise rich data obtained during the Imperial and Soviet times of our history, as many discoveries were made at that time, and many specimens were collected, studied, described, and systematised [53].

In Russia, there are currently biocollections of the following types:

(a) Biocollections created by scientific institutions or universities as a result of their research work;
(b) Nation-wide collections subsidised by governmental authorities;
(c) Museums;
(d) Biobanks [53].

They are aimed at (1) storing and providing specimens for research and (2) preserving biodiversity in Russia [53,54].

**Figure 3:**
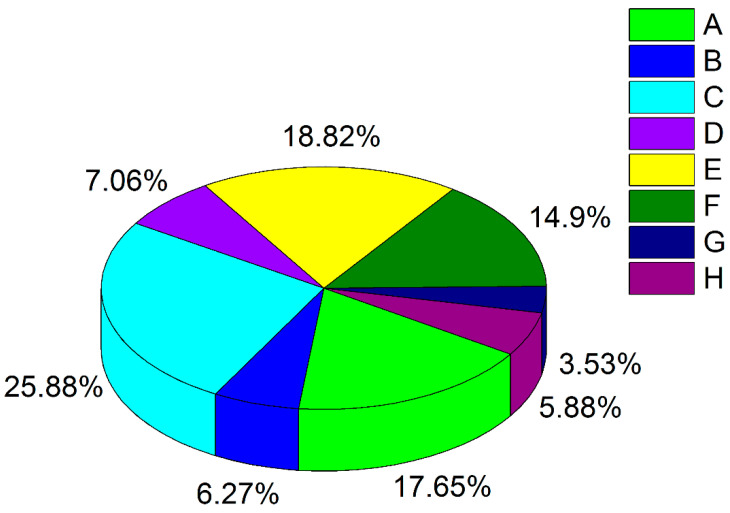
The number of documented biocollections in Russia as of May 2023 based on the data provided in [59,60]. A—microbial; B—cultures of humans and animal cells; C—agricultural plants; D—wild plants; E—herbaria; F—wild and laboratory animals; G—husbandry animals; H—biobanks.

Figure 3 summarises the information about the Russian documented biocollections that are supported by the Ministry of Science and Higher Education. There are 255 collections funded by the Ministry in total. The overall number of Russian documented biocollections has been estimated at around 280–300 [55,56].

The systematisation of Russian biocollections is at its very beginning. The information that the Ministry keeps on the biocollections contains merely the name of the collection; the institution that is responsible for managing the collection; the purpose of the collection’s creation; the number of specimens; the list of standard operational steps necessary for maintaining the collection; and the list of key infrastructure components and equipment [57].

Despite the comparatively large number of biocollections, there are currently only four national biodiversity centres:

(1) National Collection of Pathogenic Microorganisms and Cell Cultures (State Research Centre for Applied Biotechnology and Microbiology) [58];
(2) National Collection of Pathogenic Microorganisms Causing Dangerous, Extremely Dangerous and Rare Diseases of Animals (Federal Research Centre for Virology and Microbiology) [59];
(3) National Collection of Industry-Related Microorganisms (State Research Institute of Genetics and Selection of Industrial Microorganisms of the National Research Centre Kurchatov Institute) [60];
(4) National Collection of Genetic Resources of Plants (N. I. Vavilov All-Russian Institute of Plant Genetic Resources) [61].

No animal bioconservation centres are present in Russia at present.

Creating biocollection networks in Russia also seems to be in great demand. In addition to the benefits of the networks discussed above, they may significantly help in accompanying epidemiological studies, e.g., by accumulating necessary data about animal parasites, the carriers of zoonotic causative agents. This may serve to reduce the burden of zoonoses and mitigate the risk of zoonosis-related animal species extinction, as well as the risk of new human emerging infectious diseases resulting from an anthropozoonotic spillover. Therefore, this activity of biocollection networks may be of great international safety value.

### 1.4. Specific Goal

As we see from the above, in Russia, biocollections are developing, and further multifaceted work is required to expand them with the procurement of significant government and private funding. Thus, in the current work, we performed a professional opinion survey based on the opinions of Russian biologists (bioconservation specialists, big-data specialists, and ecologists). The goal was to learn their views on how to arrange, develop, and use biocollections in Russia in the most effective way.

## 2. Methods

### 2.1. Survey

To obtain a collective expert opinion on the problems of Russian biodiversity collections/centres and possible ways to solve them, we performed a survey. Our methodology is based upon the Child Health and Nutrition Research Initiative (CHNRI) approach [62,63,64,65,66,67,68,69,70]. This method allows one to detect and rank research and practical priorities in the field. In our case, this is the perspective on Russian biocollection development. The CHNRI method has been used in more than 100 published studies led by institutions and centres in the last decade [71].

Our CHNRI-based survey consisted of the following four steps:

(1) Invitation to participate in anonymous research/initial discovery of topics. Those experts who accepted the invitation to participate were asked to suggest up to three practical or research problems associated with biodiversity collections/centres in Russia. The initial invitation was dispatched to 324 persons through the social networks VKontakte, Facebook, and LinkedIn. The search for experts was carried out manually using these social networks. All of them are Russian specialists in ecology, evolution, genetics, and/or bioconservation. The selected specialists had to represent different provinces of Russia.
(2) Compilation. Identical answers were combined.
(3) Ranking. The ranking was determined in regard to five predefined criteria:
  (a) Criterion 1: Impact on the success of the research (“Do you think that the proposal will lead to new achievements in the research, facilitate the research, or open new investigation opportunities?”);
  (b) Criterion 2: Impact on biodiversity conservation (“Do you think that the proposal will stimulate bioconservation or result in finding new, more effective ways to perform bioconservation or to retard biodiversity loss?”);
  (c) Criterion 3: Impact on the promotion of “citizen science”, i.e., on elevating community involvement (“Do you think that the proposal will increase the involvement of a general population in research or practice related to biocollections?”);
  (d) Criterion 4: Potential for a paradigmatic shift in ecology and evolution (“Do you think that the proposal will or may facilitate expanding of our horizons in genetics, evolution, environmental science, or other branches of biology?”);
  (e) Criterion 5: Potential for the transfer and applicability to different practical areas (“Do you think that the results of the proposal application will be useful and usable in other areas, e.g., medicine, pharmaceutics, or different fields of biology other than bioconservation?”).
(4) Calculation. The CHNRI method involves completing a spreadsheet (e.g., rows list topics and columns criteria) with +1 (an expert supposes that a topic satisfies a criterion), 0 (an expert supposes the contrary), or 0.5 (an expert thinks that he/she has sufficient knowledge but is not inclined to answer “yes” or “no”, though this option was generally discouraged), or the field in the spreadsheet may be left blank. The response “0.5” reduces the discriminatory power of the exercise and leads to the “regression to the mean” in the final distribution of the overall Research Priority Scores (RPSs) [71].

The survey was carried out from September 2022 to February 2023.

In addition, the CHNRI methodology permits the measurement of the level of agreement among the experts for every endpoint. The indicator Average Expert Agreement (AEA) is the average proportion of scorers that returned the most common answer for a research question [70]:

$$AEA=\frac{1}{5}\overset{5}{\underset{i=1}{\sum }}\frac{N_{i,Scorers\;that\;provided\;a\;most\;frequent\;response}}{N_{i,\;Total\;scorers}},$$

where *i* runs through the set of the chosen criteria. In our case, the number of criteria is 5.

A typical spreadsheet is shown in Table 2.

For each topic and each criterion, the mean was calculated based on all answers that were not blank.

### 2.2. Software

Origin 8.1 (OriginLab, Northampton, MA, USA) was used for calculations and visualisation.

## 3. Results

Seventy-one people (21.9% of the initial set) responded with their proposals of 1–3 relevant topics. Their main demographic characteristics are provided in Table 3.

We compiled the proposed topics in one list (188 topics). Identical or similar topics were merged, giving 104 topics (55.3% of the full list of topics). Only the 24 topics (23% of the set) that passed the 5% threshold, i.e., that were suggested by four experts (5%) or more, were retained.

Then, we returned the completed list of 24 topics to each of the 71 participants to rank the priorities by scoring the topics. Of the 71 participants, only 28 (39.4% of the intermediate set of participants) responded in the second round with their rankings.

**Figure 4:**
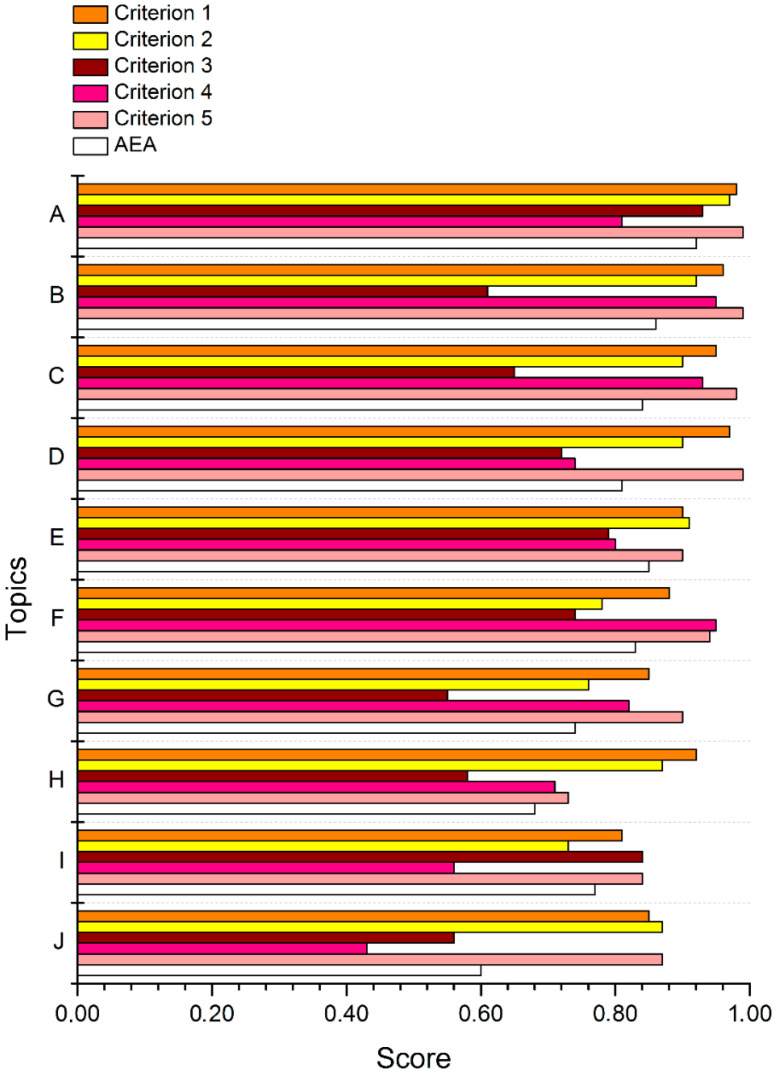
Expert scoring for the top ten proposed topics concerning the five predefined criteria along with their AEA values. AEA—Average Expert Agreement score (see Methods for details).

Figure 4 provides scores for the top ten topics suggested by the experts. Table 4 describes the topics. Ranking was performed according to the RPS values and are shown in Table 4.

## 4. Discussion

We see that the higher the rank of a topic, the greater the agreement between experts about its importance. The Pearson correlation coefficient between the RPS (Research Priority Score) and AEA (Average Expert Agreement) is 0.938 (*p* ≤ 5.9 × 10^−5^) for the top ten topics and 0.584 (*p* ≤ 7.1 × 10^−2^) for the full set of 24 topics. This means that disagreement between the experts is considerable for the 14 lowest-ranked topics, whereas the top 10 topics seem to be important to most of the experts. For example, 92% of the 28 experts who returned completed questionnaires agree with each other that there is a strict necessity to form nation-wide centres of biological diversity (topic A). Moreover, merely 51% of the experts agree with each other about the importance of ensuring the ability to perform genetic modifications of specimens in biocollections/centres (topic X).

In addition, the variance in the responses also grows as we proceed down the table of topics (Figure 4, Table 3); i.e., the discrepancy in expert opinions increases.

The top ten goals/tasks can be subdivided into three groups: (1) management-related (topics A and H); (2) science-related and technical (topics B, C, E, F, and G); and (3) policy-related ones (topics D, I, and J). Interestingly, the policy-related topics seemed important enough for the experts to place them in the top-ten group. This may be accounted for by the huge amount of paperwork that biocollection specialists ought to do in Russia now and the presence of many bureaucratic procedures that hinder them from performing scientific research and managing collections. This is in line with our previous research [72,73,74,75,76,77,78].

Although the respondents have considerable experience in working with biocollections, only 2.8 percent have published the results of their research thus far. This fact may indicate that the development of biocollections and biocollection networks is at the initial stage in Russia, and there are broad prospects for further elaboration. In addition, there are currently no uniform standards of academic writing in this field in Russia. The third reason may be insufficient governmental financing of biocollection-related investigations and the scarcity of research grants.

Thus, the surveyed Russian experts were unanimous in their opinion that the most important task for developing biodiversity collections in the country is creating large, networked, nationally important biodiversity centres, including biobanks, with free and easy access to their databases.

From the results of our research, we can deduce the main vectors for the future development of biocollections in Russia.

Many collections and samples were lost in Russia in the 1990s. The fundamental work on the preservation and development of Russian biodiversity collections practically stopped during those years and continued only due to the enthusiasm of their custodians, only resuming in 2015 when the Academician Nikolai A. Kolchanov, the former head of the Institute of Cytology and Genetics of the Siberian branch of the Russian Academy of Sciences (RAS), commenced this important activity. He attracted the attention of the authorities to the problem of the continuing catastrophic loss of national strategic resources, including animal biodiversity. The first funds for these initiatives were granted by governmental bodies only in 2016–2017. As a result, the remaining collections were renovated, rebuilt, grouped according to the objects (plants, animals, microorganisms, human materials, and cell cultures), and rated. The scientific teams responsible for the development of biocollections were granted the possibility to expand the lists of necessary equipment and material assets.

The majority of the collections were registered as the core facility centres of the corresponding organisations on the specific web resource http://ckp-rf.ru (accessed on 1 December 2023) aimed at collecting data on their operation (e.g., annual information on money spent for maintenance, money received and gained, orders, users) and providing statistics [79]. It was also designed to be a platform for the interaction between users and providers for making arrangements for the paid and unpaid use of biocollection specimens. Unfortunately, so far, that goal has not been fully fulfilled, and the work is continuing. In addition, standard operating procedures (SOPs) to maintain and expand collections and the cost calculation method of the SOPs were developed. The important tasks for the collections that were included in the new programme are expansion, the creation of specimen catalogues, and the provision of better access to users.

The N.K. Koltzov Institute of Developmental Biology RAS (IDB RAS) houses four biodiversity collections:

(1) Cell Culture Collection (CCC);
(2) Collection of the Tissues of Wild Animals;
(3) Drosophila Genetic Lines Collection;
(4) Coccinellidae Polymorphous Species Collection.

These collections have existed for many years. They are managed by the corresponding laboratories of IDB RAS, which used collection samples in its routine work and took care of them.

The IDB RAS CCC is a flagship biodiversity collection of our institution and one of the very few collections of this kind in Russia.

The first and oldest one, known as the Cell Culture Collection of Vertebrates, was founded by the Honoured Scientist Georgy P. Pinaev in the Institute of Cytology in St. Petersburg in 1978 and gathered nine collections around the Soviet Union [79,80]. Nowadays, it offers about 150 cell cultures for users and has about 800 hybridoma and patented cell cultures, which are stored according to patent requirements. The CCC, on the contrary, is a “young” cell collection, officially founded and registered in 2016, and includes more than 200 different animal and human cell cultures now (around 8000 samples). Among them are primary cultures isolated from different tissues and organs of humans and animals (laboratory and domestic animals, such as mice, rats, rabbits, pigs, monkeys, cats, and dogs, and wild animals, such as different species of hamsters and mole voles), genetically modified cells (including immortalised ones), and induced pluripotent stem cells (iPSCs) [81].

The IDB RAS CCC is a quickly developing and expanding collection. Its researchers implement the basic rules of the world’s biobanking knowledge in their routine work.

Currently, the most important tasks of the CCC are the characterisation of cell cultures (including mycoplasma tests, STR-profiling and karyotype investigations if possible, doubling time measurement, and immunocytochemistry for basic markers) and the provision of characterised cell culture samples (together with the corresponding cell culture passports) to different researchers in scientific institutions and business companies. The price of the samples provided by the CCC is very low compared with the price of the samples from known international collections, such as the American Type Culture Collection or the European Collection of Authenticated Cell Cultures (including logistics). This makes CCC materials very accessible for users and thus serves the goal of the development of Russian science and inventions in the fields of pharmaceutics, biomedicine, veterinary medicine, and gene technology.

## 5. Conclusions

We performed a survey of Russian specialists engaged in biodiversity collections. There is a substantial degree of concordance among these specialists that we need to create several nation-wide biodiversity centres. These centres will contain not only genetic material but also a variety of specimens and data, including metadata. They have to be united in a national electronic biodiversity network that will allow free access to the data stored in the centres to scientists, bioconservation researchers, and practitioners. They may significantly contribute to endangered species restoration in Russia in the future.

We have shown the success of our scientific institution in developing and managing biocollections and have deliberated upon their potential. These biocollections may become a basis for the creation of a national biodiversity centre.

## Figures and Tables

**Table 1:** Some pieces of information about three renowned international biodiversity centres.

Parameter	Bioresource Centre Riken, Japan https://web.brc.riken.jp/en/, accessed on 15 July 2023	American Type Culture Collection (ATCC), USA https://www.lgcstandards-atcc.org/en/Products/Cells_and_Microorganisms/Cell_Lines.aspx, accessed on 21 July 2023	European Collection of Authenticated Cell Cultures (ECACC), United Kingdom https://www.phe-culturecollections.org.uk/collections/ecacc.aspx, accessed on 28 July 2023
Type of funding	Government-sponsored	NGO	Government-sponsored
Number of biodiversity collections	5	5	2
Laboratory mice	9000	−	−
Plants	840,000	400 species (seeds)	−
Cell cultures	15,600	More than 20,000	Around 6700
DNA samples	3,900,000	+	−
Microorganisms	29,000	Around 20,000	+
Backup systems	+	+	+
Concordance to ISO 9001	?	ISO 9001:2015 [43] ISO 13485:2016 [44] ISO 17025:2017 [45] ISO 17034:2016 [46]	?
Digital catalogue of specimen	+	+	+
Genetic databases	−	−	−
Genome-editing possibilities	+	+	−
Sequencing possibilities	−	+	−
Other research possibilities	+	+	+
Inclusion in international projects	National Bioresource project	−	?
Inclusion in database networks	Asian collaborative networks	?	?
Educational options (undergraduate, graduate, and post-graduate studies)	+	+	+
Ethical committees	3	Adherence to common bioethical standards	?

+ Present; − absent; ? information regarding this biocollection is insufficient in open sources.

**Table 2:** An example of a completed spreadsheet that was offered for completion to 71 experts.

No. of Topic	Topic	Criterion 1	Criterion 2	Criterion 3	Criterion 4	Criterion 5
1	Topic 1	0	+1	+0.5	0	0
2	Topic 2	+1	+1	0	0	0
Etc.						

**Table 3:** Main demographics and academic profiles of the respondents detected algorithmically based on the data provided in social network accounts (if any) or self-reported. Student distribution of the sample set was assumed.

Age	Range: 26–71 Years *; Mean 36.3 ± 12.5 y.o.
Gender	28 females (39.4%)
Bioconservation specialists	23 (32.4%)
Biocollection specialists	16 (22.5%)
Ecologists	18 (25.4%)
Evolutionists, geneticists	12 (16.9%)
Other biological specialties represented	2 (2.8%)
Academic training: Bachelor’s/Master’s degree Post-graduate student PhD degree, not Professor Professor	11 (15.5%) 8 (11.3%) 46 (64.7%) 6 (8.5%)
Having considerable practical experience with starting/managing biocollections (one year or more)	53 (74.6%)
Average length of working experience in biocollections for those respondents who had practical experience	Mean 4.7 ± 0.8 years
Having published papers/books/grey literature in biocollections	2 (2.8%)

* Confidence interval 95%, *p* = 0.05.

**Table 4:** The main topics that reflect problems of biodiversity collection/centre development in Russia.

No.	Designation	Topic	RPS *
1	A	Necessity to create large national centres of biological conservation.	0.936
2	B	Full sequencing and creation of different “omic” databases.	0.886
3	C	Full digitisation of a biodiversity collection/centre.	0.882
4	D	Free-of-charge and open access to a collection/centre database for external researchers.	0.864
5	E	The presence of rich metadata in a collection/centre database.	0.860
6	F	Utilising the big-data principle for a collection/centre database.	0.858
7	G	Standardisation of specimen and data curation for simplicity of a search.	0.776
8	H	Capability of collections/centres to perform their own research on the specimens.	0.762
9	I	Free-of-charge provision of specimens to concerned parties, specifically by biobanks.	0.756
10	J	Lack of bureaucratic barriers and minimum reporting in creating, managing, and using biodiversity collections/centres.	0.716
11	K	Modelling of restoration of biogeocoenoses in collections/centres.	0.682
12	L	Identification and systematisation of pathogens causing novel emerging infectious diseases. GIS (geographical information systems) approach for the rapid identification of new niduses and outbreaks.	0.656
13	M	Using a systemic approach to creating a collection/centre. Combining data about biological associations.	0.644
14	N	Simple procedures for business entities participating in funding of collections/centres with little paperwork.	0.622
15	O	Uniting collections/centres in national networks. Using cross-referencing in all national databases.	0.618
16	P	High standards for specimen and data protection in biobanks.	0.606
17	Q	Ensuring biological safety via thorough standardisation of genetically modified organisms that pose potential threats to human and biota.	0.588
18	R	Increasing the government funding of biocollection/centre development.	0.562
19	S	Inclusion of cultural- and region-specific metadata on agricultural, food, veterinary, or medicinal use of specimens.	0.512
20	T	International cooperation. Inclusion of national collections/centres in international projects, initiatives, and networks.	0.506
21	U	Relevant legal support of collection/centre functioning. Development of corresponding laws.	0.418
22	V	Simplifying the patent work in collaboration with collections/centres.	0.362
23	W	Integration of biodiversity collections/centres with educational programmes.	0.306
24	X	Ability to perform work on genetic modification of specimens.	0.248

* RPS—Research Priority Score (see Methods for details).

## Data Availability

Available from the authors upon reasonable request.
